# Comparison of systolic and diastolic criteria for isolated left ventricular noncompaction in cardiac MRI

**DOI:** 10.1186/1532-429X-14-S1-P148

**Published:** 2012-02-01

**Authors:** Brandon Stacey, William Hundley, Vinay Thohan

**Affiliations:** 1 Cardiology, Wake Forest University School of Medicine, Winston-Salem, NC, USA

## Background

Interest in left ventricular non-compaction (LVNC) as a distinct clinical form of cardiomyopathy is supported by recent publications. Echocardiographic and select cardiac MRI criteria have been established which are used to facilitate the diagnosis and have led to concerns of diagnostic accuracy. We used cardiac magnetic resonance imaging (cMRI) to assess standard criteria for LVNC.

## Methods

Trabeculation/ possible LVNC by cMRI was retrospectively observed among 122 consecutive cases and comprised our study population. We compared standard end systolic (ES) and end diastolic (ED) criteria previously established along with myocardial thickening (MT), ejection fraction (EF), 3D sphericity index (3DS), and LV end-diastolic volume index (EDVi). Using analysis of covariance, adjusted means for EF, MT, and 3DS were generated by adjusting for age, race, gender, body surface area (BSA), diabetes mellitus, hypertension, hyperlipidemia, coronary artery disease, and congestive heart failure (CHF). Adjusting for these same covariates except CHF, logistic regression was used to compare the odds of CHF for those who met criteria for NC at ES vs. those at ED.

## Results

ES noncompacted: compacted ratio (NCCR) had stronger correlations with MT (-0.49), EF (-68), EDVi (0.63), and 3DS (0.55) than the ED NCCR (MT: -0.22; EF: -0.25; EDVi: 0.37; 3DS: 0.32). After adjustment for covariates, those who met criteria for NC by ES NCCR had a lower EF (p=0.01) and less MT than those who did not (p =0.01 and p=0.003, respectively), but there was no statistical difference in EF or MT between those who met criteria for ED NCCR and those who did not. The odds ratio of CHF for those who met ES NCCR criteria was 29.4 (CI: 6.6-125), but the odds ratio of CHF for those who met ED NCCR criteria was 3.3 (CI: 1.1-9.2).

## Conclusions

ES measures of NC have stronger associations with systolic dysfunction and CHF than the ED measures.

## Funding

No funding source used for this project.

**Table 1:** Comparison of Ejection Fraction and Myocardial Thickening between End-Systolic and End-Diastolic Criteria for Noncompaction in Cardiac MRI

Measure	Ejection Fraction (%)	P-Value	Myocardial Thickening(%)	P-Value
ES NC:C Ratio > 2	31.8 ± 6	0.01	9.4 ± 16.2	0.003
ES NC:C Ratio < 2	40.4 ± 8.8		37.2 ± 12	

				

ED NC:C Ratio > 2.3	36.4 ± 5.4	NS	37.3 ± 12.3	0.07
ED NC:C Ratio < 2.3	38.4 ± 4.5		27.0 ± 14.8	

Analysis of covariance used to generate adjusted means with 95% confidence interval. Adjusted for age, race, gender, BSA, diabetes mellitus, hypertension, hyperlipidemia, coronary artery disease, and congestive heart failure.

**Table 2:** Odds Ratios for Congestive Heart Failure by Noncompaction:Compaction Ratio

Measurement	Odds Ratio (95% Confidence Interval)	P-Value
End-Systolic Noncompacted:Compacted Ratio (Continuous Variable)	20.9 (5.7 - 76.7)	< 0.001
End-Diastolic Noncompacted:Compacted Ratio (Continuous Variable)	3.3 (1.4 - 8.0)	0.006
End-Systolic Noncompacted:Compacted Ratio > 2	29.4 (6.6 - 125)	< 0.001
End-Diastolic Noncompacted:Compacted Ratio > 2.3	3.3 (1.13 - 9.2)	0.028

Logistic regression analysis used to generate odds-ratios for congestive heart failure. Adjusted for age, race, gender, BSA, diabetes mellitus, hypertension, hyperlipidemia, coronary artery disease.

